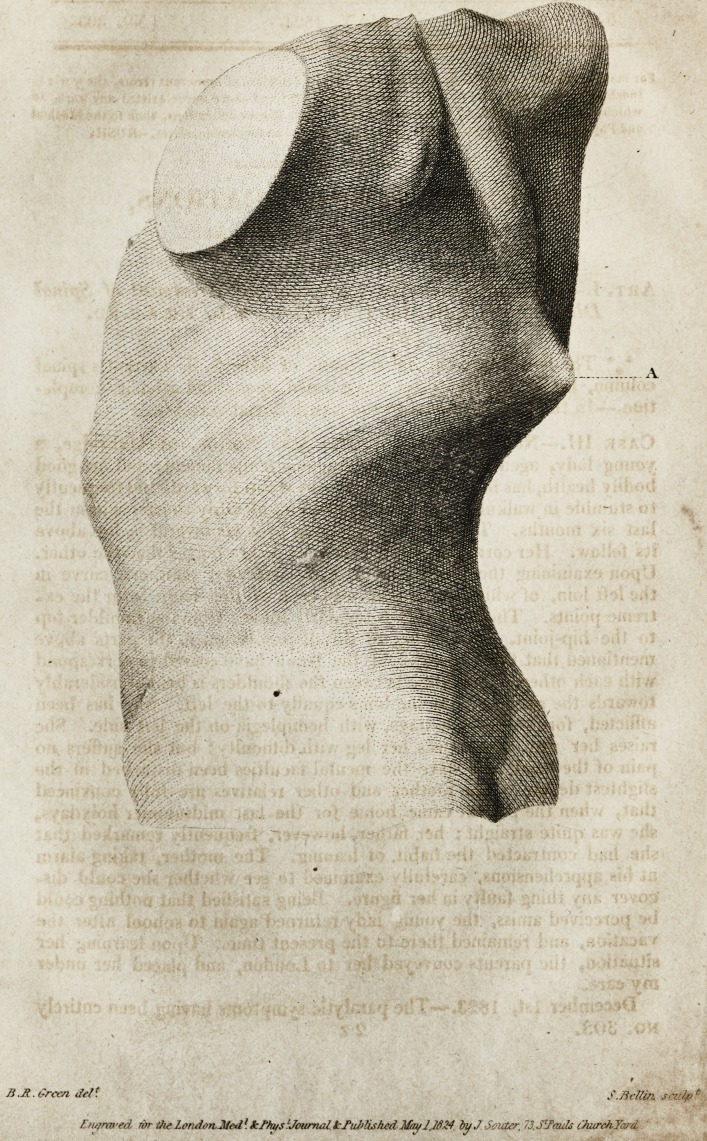# Observations on the Pathology and Treatment of Spinal Diseases

**Published:** 1824-05

**Authors:** Edward Harrison


THE LONDON
### Medical and Physical Journal.

5 OF VOL. LI.] MAY, 1824. [No. 303.

For many fortunate discoveries in medicine, and for the detection of numerous errors, the world is indebted to the rapid circulation of Monthly Journals; and there never existed any work, to which the Faculty, in Europe and America, were under deeper obligations, than to the Medical and Physical Journal of London, now forming a long, but an invaluable, series.—RUSH.

### ORIGINAL COMMUNICATIONS,
### SELECT OBSERVATIONS, &c.

ART. I.—*Observations on the Pathology and Treatment of Spinal Diseases.* By EDWARD HARRISON, M.D. F.R.A.S. ED.

[With Engravings.]

*** The two Prints explain the state of Miss S. S. Tarrant's spinal column, before the treatment was entered upon, and after its completion.—In the first, A points to the eighth dorsal vertebra.

CASE III.—November 26th, 1823.—Miss Norton, of Uxbridge, a young lady, aged fourteen, of a sanguine temperament, and in good bodily health, has been observed lately to stoop forward, and frequently to stumble in walking. She has grown considerably shorter within the last six months. The right shoulder-top projects several inches above its fellow. Her corresponding leg is three inches longer than the other. Upon examining the naked person, I discovered a semilunar curve in the left loin, of which the scapula and crest of the ileum form the extreme points. The right side is regularly convex from the shoulder-top to the hip-joint. It is owing to this disproportion in the parts above mentioned that the two sides of the trunk have ceased to correspond with each other. The spine between the shoulders is bent considerably towards the right, and in the loins equally to the left. She has been afflicted, for the last few days, with hemiplegia on the left side. She raises her arm and moves her leg with difficulty; but she suffers no pain of the head, nor have the mental faculties been disturbed in the slightest degree. The mother and other relatives are fully convinced that, when the patient came home for the last midsummer holydays, she was quite straight: her father, however, frequently remarked that she had contracted the habit of leaning. The mother, taking alarm at his apprehensions, carefully examined to see whether she could discover any thing faulty in her figure. Being satisfied that nothing could be perceived amiss, the young lady returned again to school after the vacation, and remained there to the present time. Upon learning her situation, the parents conveyed her to London, and placed her under my care.

December 1st, 1823.—The paralytic symptoms having been entirely removed by rest, the exhibition of saline aperients, and the antiphlogistic regimen, she this morning submitted to the usual treatment for the cure of her spinal complaint. The chest and back were well rubbed for more than an hour; during the whole time her person was stretched in the usual machine, *(for a description of which see page* 354.) A broad bandage was then drawn tight round her, and fastened in that situation with buckles. Afterwards she was laid flat upon an horizontal crib, and directed to remain constantly upon her back.

December 5th.—The rubbing and stretching have been daily repeated for the period recommended; at all other times she has worn the bandage, and lain upon her back. The spine having nearly recovered its natural situation, the shoulders, hips, and feet, are almost equal.—Continue.

December 11th.—The only remaining defect in the spinal column is a little elevation of the lumbar vertebræ. There is no perceptible difference in the appearance of the shoulders, hips, or feet.—Continue.

December 16th.—A wooden shield, properly constructed, was this day placed upon the back under the bandage, to produce regulated pressure upon the spinal column, from the top of her back to the sacrum.

December 21st.—The protuberant vertebræ having entirely disappeared, and there being no longer any defect in the spine, the treatment is to be discontinued. Her health is in all respects good.

Miss Norton being at a considerable distance from home when the complaint first appeared, recourse was had on the occasion to a medical practitioner in the neighbourhood, and afterwards to the family surgeon. Both these gentlemen deeming her recovery impracticable, disapproved of every attempt for that purpose. In this advice they were zealously seconded by an affectionate uncle, who, having made inquiries among the faculty of his acquaintance, was led to consider her restoration impossible; and, moreover, that the health would probably sustain further injury by the trial. The anxiety of her parents could not be restrained; they determined to leave no effort unattempted for the recovery of their daughter's person and health. In little more than a fortnight after her arrival in London, the uncle called, and, having carefully surveyed his neice from head to foot, declared, unless he had witnessed the alteration with his own eyes, he could not have been prevailed upon to give credit to the improvement which he saw. Besides deformity, she had the misfortune, at my first visit, to be afflicted with paralysis of the arm and leg. The latter complaint was very recent, and probably arose from pressure upon the axillary and crural nerves near to their spinal ends. I have been led to this conclusion from having observed the deleterious effects of pressure upon the spinal nerves in many other instances, and because the functions of the brain were not in the smallest degree disturbed, either before or during the seizure.

Though it may be difficult to fix upon the exciting causes, the predisposition was doubtless laid in general debility. The rapidity of the cure shows with how much ease and safety recent cases admit of removal: it is, therefore, very desirable that recourse should be had to medical advice on the first appearance of indisposition; otherwise, the malady will not only increase in virulence, but become more obstinate from delay. I will venture to assert, when taken in an early stage, few complaints are of easier or more certain cure than this variety of spinal disease.

IV. *A Case of Paraplegia.*—October 28th, 1823.—Mrs. Wells, of Hemel Hempstead, aged forty, had the misfortune to be thrown out of an open carriage with great violence, upon her face and elbows, some time in August 1817. She was much bruised, but felt no particular inconvenience till March 1819. During this time her shoulders were observed to grow more prominent, and to be accompanied with a marked depression in the upper part of her chest. She has been a severe sufferer ever since, and has taken many medicines of different kinds, without obtaining the smallest relief from any of them. The case was for a long time supposed to be rheumatic, and treated accordingly. In the spring of 1820, she consulted a very experienced and highly respectable surgeon: he said that she was suffering under a disease of the spine, in which several vertebræ *were decayed,* and added that nothing could be advised for her with the smallest prospect of relief. He recommended, amongst other things, the insertion of caustic issues near to the part most protuberant. His directions were neglected, owing to the despondency of her medical attendant; blisters being the only applications tried.

Has little use of the upper, and none of the lower, limbs: they always feel heavy, cold, and numb, though to other persons they are sometimes cold, at other times hot. She is unable to bend her back in the smallest degree; nor can she turn herself to either side, as she lies in bed. The weakness of her limbs and back came on imperceptibly, and have increased very slowly. It was more than two years after the accident occurred, before she was entirely deprived of all power, in either the back or inferior extremities. Appetite has lately declined, and her liver has often been affected. The latter complaint is discovered by looking at the countenance, which is at those times dull, sallow, and of a yellowish brown. The urine is frequently turbid, with an ammoniacal smell; motions dark-coloured and offensive; menses are of a brown or greenish hue, and emit a disagreeable odour; pulse weak, and of moderate frequency. All the dorsal vertebræ and ribs, by their extraordinary prominence, form an unsightly convexity, or gibbous swelling. The third, fourth, fifth, and sixth, in particular, rise considerably above the rest, forming the arc of a small circle, and their spinous eminences bend a little towards the left. Owing to this partial turn, the transverse processes may be distinctly felt on the right side. The dorsal convexity has produced a disagreeable hollow in the small of her back, with a corresponding fulness of the abdomen. There is not, and never has been, tenderness in any part of the spine.

The patient, being placed upon a couch, as usual, had her back and chest well rubbed with an emollient liniment for more than an hour, while the spine was stretched in the machine formerly referred to. It is constructed of steel, upon the principle of the windlass of a ship, and fixed to the bottom of the crib. By means of soft leathers surrounding the arms, and connected with the top of the couch, and other leathers attached to the ankles, which are fixed to the machine, almost any degree of stretching may be safely resorted to, by turning the roller of the machine, provided the force be gradually increased. The prominent vertebræ and ribs were then pressed, and driven in the direction of their natural situations, with an instrument held in the right hand. It has a wooden handle, into which is fixed, at right angles, a brass rod, four inches in length, and of strength enough to bear any degree of force that the operator may deem it prudent to apply. To the lower end another round piece of brass metal, about two inches long, is rivetted at right angles. This, well covered with soft leather, to prevent its bruising the skin, constitutes, with the other parts, the instrument that I employ in all my manipulations. I formerly used my thumbs only for pressure; but, finding the other contrivance much more powerful and easier to be borne, I have for a long time given it the preference. A firm bandage was afterwards fastened round the chest, to prevent the bones from returning. This bandage being adjusted, she was laid flat upon the back, and directed to remain constantly in the same position. The patient's dress, and divisions of the mattress, admit of all the natural offices being conveniently performed without moving the trunk of the body.

November 4th.—The arms and legs have acquired more feeling, a better colour, and their natural warmth. She can already move her arms freely, and her toes a little. The back is less arched; only two of the four irregular vertebræ appear above the rest. The means of cure above recommended have been daily repeated since last report. A steel shield was this day placed over the most prominent vertebræ, and under her bandage, to confine them and make stronger pressure.

November 16th.—The contour of the spine is entirely restored, with the exception of the fourth and fifth dorsal vertebræ, which still project a little; their transverse processes continue partially depressed, and turned towards the left side. Those of the right may be easily felt. She has recovered the faculty of moving her hips, knees, ankles, and toes, with some freedom in both limbs. The left leg has from the first been weaker, and continues more infirm than its fellow.

November 28th.—Besides bending the lower extremities with greater facility, she can now draw them upwards, and push them back with some degree of force.

December 8th.—Every day since last report has been marked with increased ability of the lower extremities; she moves them with great freedom and ease in every direction. The proper form of the back is entirely restored, except where the two vertebræ above mentioned have raised the skin into a small circular tumor.

December 10th.—The patient continues in excellent health, has a good appetite, and sleeps well. She moves her limbs with facility in every direction. She can not only pull them up, and push them down, but crosses and draws them back again, without the smallest difficulty.

December 31st.—The strength and activity of the lower extremities have lately increased. She can elevate and depress her chest and belly with great ease. The projecting vertebræ are lower. Her countenance is much improved.

January 28th, 1824.—The two vertebræ formerly described still remain slightly raised. In all other respects the spinal column has recovered its lost figure. The menses and urine have regained their natural properties. She is in excellent health and spirits. She was this morning assisted from her couch, and walked about in the room for ten minutes with great ease, and strength of her lower limbs.

January 30th.—She was permitted to return home this day, on condition that she agreed to maintain the horizontal posture until the ligaments of the spine had entirely recovered their lost tone and vigour.

March 17th.—She has enjoyed good health since her return home, and looks better. Her back has gained strength, and is rather flatter. She feels stronger in her limbs, but has remained constantly on her couch.

Mrs. Wells had a young child in her arms at the time of her accident, and contrived to preserve it unhurt. She was too much terrified to know exactly how she fell, but thinks it was either upon her side or upon her elbows and face. She sustained so little inconvenience at the moment, that, from the date of her fall till March 1819, an interval of nineteen months, she followed the laborious occupation of a baker. The neighbours remarked that she slowly lost her natural figure during this period, though they were unable to account for the change produced in her person. I am inclined to believe that, although she did not tumble upon her back, she distressed it, and strained the ligaments, by her efforts to defend the child and herself. Had she abstained from work only a few days, the strained parts would probably have recovered their tone, and no disadvantage have resulted from the accident. Instead of using the necessary precautions, she continued to employ her hands in working the dough, and, after it was baked, in tossing the loaves into a higher apartment for sale. By these daily occupations, she gradually weakened the spinal ligaments between her shoulders, which had sustained a shock from her fall, and they, no longer able to confine the vertebræ, suffered them to slide outwards. The ribs followed, in consequence of their firm connexion with the spinal column. Had the vertebræ been ulcerated or injured in their structure, every effort to restore the spine and ribs would have been more detrimental than beneficial. It is evident, from a careful regard to the symptoms, and the successful termination of the case, that the vertebræ were displaced, but not *decayed*, though the latter had been asserted on very respectable authority, and by a most honourable practitioner.

The foregoing explanation derives consistency and support from the following communication:—Three stout healthy sisters contracted severe spinal complaints, as it was believed, in the following manner. The mother, an athletic woman, used to amuse herself by alternately tossing them aloft, and catching them under the arms. She spent some hours daily in this diversion. The backs of her children received so many jars and twists in the prosecution of these rude sports, that the ligaments became stretched and weakened, till they were no longer able to keep the vertebræ fixed and stationary; they accordingly gave way, and spinal distortion followed.

Case V.—September 15th, 1820.—Miss D. J., aged twelve years and a half, of the sanguine temperament, with a soft skin and fair complexion, is suffering from lateral curvature of the spine. It passes under the right shoulder-blade, and is very considerable. The scapulæ at their lower edges nearly touch, and she can strike them together with great ease and force: the right is most prominent. She has an opposite curve in the lumbar spine. The ribs in the right hypochondre are thrust down; in the left they stand prominently forwards, making an oblong swelling in front. The chest is peaked, and the left shoulder-top has sunk below its fellow. The countenance is contracted; it looks aged and care-worn. She is become very inactive and desponding, under a strong apprehension that her malady is incurable; many gentlemen of the faculty having given it as their decided opinion, that nothing could be undertaken with the smallest expectation of removing it. The complaint is of three and a half year's duration. Menses appeared, for the first time, four days since.

This young lady, being placed upon her crib in the manner directed in other cases, had her chest and back well rubbed for an hour. The vertebræ and heads of the ribs were afterwards forcibly driven in the direction they had left. During the whole time her spine was stretched and drawn out, but less effectually at this early period of the treatment. It was done by one person pulling at the arms, while another drew out the legs and feet in an opposite direction. The usual bandage being placed round her chest, she was ordered to remain constantly flat upon her back.

October 21st.—The process above mentioned has been repeated daily, except the manual pressure, which is only applied every other day. A wooden shield, adapted to her back, and contrived so as to rest against the most projecting parts, being fastened under her bandage, she was put into the usual position. The lumbar curve is nearly effaced; the other is already much fainter; and the scapulæ are separated to a greater distance from each other. The depression of the ribs is less. The oblong ridge in front has nearly disappeared; the ribs on the right side are risen higher; the chest looks rounder, and more expanded.

November 13th.—The features have recovered their natural aspect, and the health is particularly good. The lumbar vertebræ are entirely rectified. The front ribs on the left side are quite restored; those on the right are more elevated. She looks better in every part, and the spine is become nearly straight. Menses re-appeared within the last fortnight.

March 10th, 1821.—The spine and ribs have returned to their natural situations; the form of the trunk, which was originally very fine, is entirely restored. She looks extremely well, is in excellent spirits and good health.

June 5th.—The patient is plumper, and considerably taller since she adopted the reclining posture. Her face is become florid, open, and juvenile; she preserves her excellent figure and good health. Appetite, menses, and bowels, are all regular.

*First Plate.**—A, B. Right and left shoulder-tops. C, D. Right and left hips. E. Hollow in the left loin. F, G. Spinal curvatures to the right and left. H. Prominence on the right side. I. Ditto on the left side.

*Second Plate,* taken when the treatment was concluded.

Soon after her convalescence, this young lady retired to the continent, and has visited several places of fashionable resort. She is an attractive belle in all companies, and particularly distinguished for the symmetry of her person.

An opinion seems to prevail, which it will be proper to notice in this place,—viz. that fibrous structure, being incapable of stretching, never contributes towards the spinal gibbosities, incurvations, and monstrous deformities, which are so prevalent among delicate persons. How far this opinion rests upon a firm basis, will be more clearly understood when we have taken a survey of the several parts which compose the vertebral joints. They may, for this purpose, be divided into, first, the white, and secondly, the yellow ligaments.

1st. It has been observed by ancient and modern writers, that the humerus and maxilla inferior sometimes fall out of their sockets, through weakness of the ligaments.† I have myself witnessed a similar accident in the wrist of a young man. The fractured portions of the os femoris, when the misfortune takes place within the capsular ligament, are seldom re-united. The whole limb in such cases grows gradually shorter, by the superior foot pushing upwards, and rising above the acetabulum. This alteration is necessarily attended with elongation of the

* These Plates are unavoidably postponed. Our readers will find those of Miss Tarrant's case in the present Number. In binding up the volume, they can be placed in their proper situations.

† Celsus.

capsular ligament, the tendons, and other component parts of the hip-joint. Unless this change took place, it is clear that no new arrangement could be made in the situation and disposition of the thigh. As these accidents are produced by elongation of white articulating fibres, it follows that these substances will stretch, under certain favourable circumstances. The several joints mentioned have, like the spine, their capsular ligaments, their tendinous cords, and membranous expansions, by which the moveable bony extremities are firmly supported and preserved in close connexion. So long as this tissue maintains its tone unimpaired, the ends of the bones remain in contact. It is only in proportion to the relaxation and stretching of this substance, that they are disjointed and severed from each other. Though none of these membranes will admit of sudden extension without being injured, they may be safely, and often are, considerably elongated by a slow and gradual progression. It is in this manner that the vertebral joints give way to the regular and imperceptible operation of constitutional causes.

2d. All yellow ligaments are elastic and extensible to a great degree, as we see in the suspensory ligaments of the heads of quadrupeds, in the substances placed between the cartilaginous rings of the trachea, in the middle coat of arteries, and in the outer membrane of the corpora cavernosa penis. The yellow ligaments of the spine are strong, flexible, and admit of considerable motion. They, in the first instance, more than the white, suffer the vertebræ to vacillate and recede. Afterwards both structures are brought to participate, and then the disorder advances with greater regularity and rapidity. All the vertebræ are articulated and possessed of motion, but it is most limited in the dorsal vertebræ.

An original difference is observable in the flexibility of the joints in different persons, which dispose certain individuals, more than others, to suffer from spinal maladies. Some adults, in the possession of good health, are endowed with such extraordinary faculties, that they can strike their finger-ends against the back of their hands; they can also bend their bodies so as to hit the hind head with their heels, and put the great toe of either foot within their mouths. I have seen a person thrust his legs backwards as far as the knees over his shoulders, and, having grasped them above the ankles, suffer himself to be rolled over like a ball. Unless the spinal ligaments were, in all these instances, endowed with great elasticity, they could not act in the manner described. We may therefore conclude that, in these instances, the several ligaments which fasten these joints to one another, admit of being considerably stretched, and pulled, and bent, without being either torn or injured.

The foregoing observations apply directly and forcibly to explain those deviations of the spine, in which the white and yellow ligaments are equally concerned. If we admit, as we must necessarily do, the elasticity of these substances, and take into account their anatomical connexion with the vertebræ and with one another, we shall find no difficulty in understanding by what agency the vertebræ slide out of their places, and form those monstrous distortions which daily meet our eyes in the crowded streets of this great capital. They may be produced in the most healthy constitution, by adopting a leaning posture, and continuing in it day after day, for a long time together. This is exemplified in the colliers of a particular mine in Lancashire, who are, I am told, obliged, from the thinness of the stratum, to sit in a leaning posture, and force the right side into the vein, while digging out the coal. In process of time the spine is, in all of them, curved towards the right, from their continuing so many hours in a leaning attitude. These labourers sleep above ground, and therefore preserve their health unimpaired, except in as far as it suffers from their mode of working among the coal. Similar gibbosities, arising from the same cause, are said to prevail among the colliers near Rotherham, in Yorkshire. The intervertebral substance is in these persons squeezed together, and compressed on the left side, by the bodies of the vertebræ being pressed nearer to each other. The same vertebræ are forced asunder on the opposite side: in consequence, the ligaments are stretched and extended. When the position is changed, and the incumbent weight removed, the intervertebral matter for some time recovers its former dimensions. The vertebræ no longer press unduly upon the ligaments, which contract by an inherent elasticity, and resume their former expansion. Repetition produces similar effects, which are also removed in the same manner. At length some of the ligaments give way, and permit a single vertebra to jut out. A lodgment being once made, and the same causes continuing to operate, it protrudes more and more. Other vertebræ follow in succession, because, the proper support being taken away, they no longer receive their accustomed protection.

A knowledge of the injurious effects of posture upon the spines of hardy labourers cannot be too strongly impressed upon the minds of parents and teachers. An over anxiety to educate girls in the fascinating accomplishments of music, drawing, and dancing, by which they are confined in hot rooms, and put into strained attitudes for hours together, has led, in different ways, to the most distressing consequences. If long perseverance in any habit be sufficient to produce distortion and deformity in the spinal arrangement of adult and athletic males, it will be much more likely to induce them in the sickly and pampered children of the affluent. Though various causes may be assigned for the increased prevalence of these complaints in our time, I am convinced that the relaxing effects of hot rooms, and a too-ardent pursuit after feminine accomplishments, are the principal ones. By admitting the elasticity of fibrous structure, and its disposition to stretch under certain favourable circumstances, we are enabled to understand many obscure actions which are going on in the animal economy, and, in particular, to fix spinal distortions upon a simple and stable foundation.

Having introduced the foregoing observations, I proceed to give a brief anatomical description of the various matters which constitute the spinal joints. The vertebræ are chiefly preserved in their natural situations, first, by an intermediate fibro-cartilaginous substance, which is compressible, elastic, and extensible; secondly, by the ligamenta antica et postica vertebrarum communia; thirdly, by the ligamenta intervertebralia; and, lastly, by the strong yellow interspinous ligaments. Besides this complicated apparatus, the vertebral column is further defended, and its motions limited, by the articulations of the transverse processes.

These are the organs which immediately surround the vertebræ, maintain them in their places, and, by their elasticity, enable posture-masters, tumblers, and mountebanks, with the assistance of the connected muscles, to perform their different contortions, and exhibit those preposterous attitudes, backward, forward, and sideways, which astonish the spectators. Were any of the substances concerned in these movements either inelastic, inflexibly rigid, or incapable of being readily stretched to a certain degree, they would burst asunder, or be otherwise so much hurt as to lead to the most lamentable consequences.

It follows, from the preceding observations, that the opinion which prevails generally in this country, of the spinal column being too firmly joined together to admit of the smallest separation, under ordinary circumstances, rests upon an untenable foundation. Indeed, this conclusion is opposed to every day's experience of what is going forward in the animal economy.

The flexibility of the spinal chain was well understood and fully admitted by the Father of Physic, as appears by the expressions that he employed when treating of it. The term ϱαχις, *spina dorsi*, is derived from ρησσω, *frango, quia spina multis vertebris rupta.* He also denominates the spine κλονιον, from κλονεω, to shake or tremble. It is clear, from the words used, as well as from the tenor of his numerous writings, that Hippocrates was well acquainted with the disposition of the spine to lose its natural arrangement, both from hurts and constitutional causes.

After the foregoing remarks on the extensibility of fibrous structure, I proceed to consider more particularly the nature and treatment of spinal complaints. Their alarming increase, their extraordinary obstinacy, and the many distressing consequences to which they lead, entitle them to the attentive consideration of all medical practitioners. These maladies are, as I have already observed, produced in various ways, and fix their seats in different tissues; but, as an elongation of the ligaments, with displacement of the vertebræ, is the most common cause, I propose, in the first division, to confine myself to this variety.

Luxation, or dislocation,* is said to have taken place when the natural relation subsisting between two articulating bones is interrupted; or, in other words, when the moveable surface of one ceases exactly to correspond with its fellow.

There are two sorts of luxation, the complete and the incomplete. The former requires the articular extremity of one bone to be wholly driven from the corresponding surface of the other.† In the latter, the articulating extremities are only partially separated, not entirely disjoined; a greater or smaller extent of the displaced bone still remaining in contact with some portion of the other.

It follows, from this statement, that many joints admit of every degree of luxation, from the slightest parting to complete disunion. In all, the articular motions are imperfectly performed, because the surfaces of the bones do not fully correspond.

It is a general opinion that the cervical and lumbar vertebræ admit of complete dislocation, from the application of external violence. The dorsal vertebræ are supposed, by modern practitioners, to be too firmly connected with the ribs, and with each other, to allow of luxation from any cause. In this conclusion they are directly opposed to the writers of antiquity. CELSUS, in the chapter *de Spina Luxata*, observes,‡ "Excidunt autem vertebræ et in posteriorem partem, et in priorem, et supra septum transversum et infra." HIPPOCRATES§ treats of luxations at great length, in the section *de Articulis*. Whenever the vertebral joints are suddenly burst asunder, which he says

---

* In medical writings, dislocation and luxation are terms of the same import. The former derives its origin from *de loco*, out of place; the latter from *luxo*, to loosen or disunite.

† Maxilla, vero et vertebræ, omnesque articuli, cum validis nervis comprehenduntur, excidunt aut vi expulsi, aut aliquo causu nervis vel ruptis vel *infirmatis*: faciliusque in pueris et adolescentulis, quam in robustioribus.—CELSUS *de Ossibus Luxatis*.

‡ See CELSUS *de Medicina*, Lib. viii. cap. 14.

§ HIPPOCRATES *de Articulis*, sec. 6.

they may be in any part of the spinal column, the connecting fibres are always ruptured, and the vertebræ generally fractured.

I was consulted, several years since, for F. G. Pratt,* who had suffered more than nine months with a dislocated lumbar vertebra. It was of the species described by Celsus, in which the vertebræ "toto loco motæ sunt." I only saw the boy twice. He was brought to me the first time. On calling a few days afterwards, I recommended to his father an examination of the diseased parts, when an opportunity offered; from which it is to be inferred, that I thought something would be disclosed by a post-mortem investigation. I moreover declared that, together with dislocation, some of the ligaments would be found broken, and the nervous substance injured. I said nothing about fracture of the vertebræ, as has been erroneously asserted. The tract of the lumbar spine lies so deep, that, after a lapse of nine months, it would, I conceive, have been next to impossible to come to a satisfactory decision upon such a question. I might have justly laid myself open to the imputation of ignorance and presumption, had I made any attempt, under these hazardous circumstances, to remove the luxation. Such was my conviction of its severity and fatal tendency, at the first interview, that I positively refused, both then and afterwards, to interfere professionally, though urgently importuned by the mother to afford my assistance.

2d. The other species of luxation, under which the joints occasionally suffer, has been unaccountably overlooked in modern times, though it was well understood by the ancients. Many interesting facts and remarks lie dispersed in their writings, and the subject is treated at considerable length by Hippocrates and Celsus. In complete dislocations, the articulating extremities, as we have already observed, are entirely disjoined; whereas, in sub-luxations,† they are partially severed, not wholly disunited. In some, the joints merely gape. "Nam modo, quo juncta sunt, inter se dehiscunt." In others, particularly the vertebræ, they sink down, or rise a little out of the line. "Nonnunquam enim nervorum imbecillitas efficit ut quamvis non exciderit vertebra, paululum tamen in priorem partem promineat."‡ Every degree of dislocation, which does not amount to complete separation, is properly a sub-luxation. It follows from this statement, that, in the same joints, the dislocations vary considerably in extent and magnitude. The tumor in one part and cavity in another differ, also, according to the degree of luxation. The accompanying lameness will

* See *London Medical and Physical Journal.*

† Morgagni, *Epist.* lvi. art. 35.

‡ Celsus.

likewise generally bear a proportion to the disunion. It becomes a matter of very great importance, on every occasion, to discover, without delay, the true nature of all hurts, and determine whether they have produced dislocation or are merely bruises; because the mode of cure, to be successful, must be conducted accordingly.

I have at present selected only five cases of deformity out of many similar ones, thinking them amply sufficient to elucidate the pathology, and establish the superior efficacy, of my treatment. To prevent the danger of further perplexity and misconception, I have added the names and abode of the persons mentioned in the narrative. Several others, equally afflicted, might here be introduced, who are at this time largely participating in the amusements of London. The two Prints, taken from the first models, show the deplorable condition to which the patients were reduced before the process began. These, and the corresponding engravings, illustrate the astonishing renovation which followed the methods employed for that purpose. No similar trials, as far as my information extends, had ever been attempted, or even suggested, before I ventured to hazard the experiments: that they were completely successful, cannot, I think, be denied. Should further proofs be desired, I will with pleasure refer the inquirer to many who have been equally benefited, and are now filling useful stations in society.

Had the vertebræ or cartilages ever been unsound, the patients, instead of deriving relief, would, I conceive, have suffered greatly, from the practice employed. No osseous union could have formed between the vertebræ, to obstruct the process of cure. Such an impediment would have proved an insurmountable barrier, and defeated the whole plan. The gibbosities in the two first proceeded from displacement of the vertebræ and ribs at their spinal ends. Had the malady commenced with inflammatory symptoms, osseous matter would probably have been deposited between the vertebræ in sufficient quantities to convert them into one mass. When the malady arises from pure relaxation, the neighbouring vessels are not roused into inordinate action, or encouraged to secrete a superabundance of bone. It may be truly said that the protuberant vertebræ had, in both instances, been reduced to a state of anchylosis, or crookedness, not because they were consolidated by osseous materials, but because they were distorted and their motions lost. Anchylosis is frequently used in this latter sense by the ancients: it is derived from *αγκυλος*, *curvus*. The immobility was owing to vertebral luxation, and in this respect resembled the rigidity that occurs in other dislocations of long standing. The removal of the gibbosities was of necessity tedious, partly because they had subsisted for a long period, and partly because it would have been dangerous to hurry the process, where so much required to be done.

With the third patient, the disease being of recent origin, there was nothing to apprehend from expedition, and therefore the cure was speedily completed.

The fourth was a case of paraplegia. It arose from pressure upon the spinal cord, in consequence of the dorsal vertebræ being forced out of their proper situations, and was progressively removed, as they were driven back again, by the process adopted for that purpose, and which I have detailed above. The increase of sensibility and power of motion in the lower extremities kept pace with the spinal improvement. According to my experience, the seat of paraplegia is always in the back. Compression in the upper part of the spinal cord affects the arms. Wherever it is fixed, the lower extremities are found to suffer; when slight, the oppressed parts feel weak, numb, and cold. They are also subject to irregular cramps and distressing spasms, first in one part, and then in another. Stronger pressure occasions immobility, and entire loss of sensation. Such was the deplorable condition to which Mrs. Wells was reduced when I first saw her, and from which she was happily rescued by the means resorted to for her cure.

The last young lady, whose case has been described, is so completely recovered, by the process adopted, from hideous deformity and the *precursory symptoms of pulmonary consumption*, that she has become successively the beauty of Spa, Brussels, and other places.

[To be continued.]

## Figures and Tables

**Figure f1:**
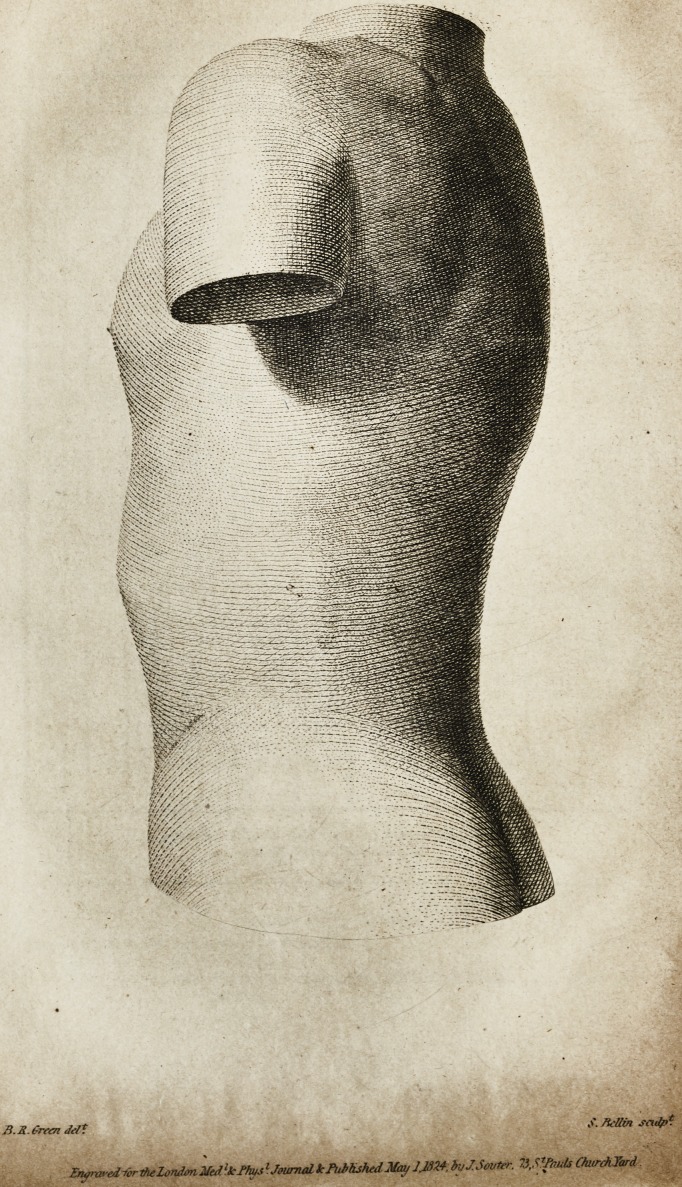


**Figure f2:**